# DNA lipid nanoparticles as alcohol-sensitive surrogates to trace microbial transmission and monitor hand hygiene

**DOI:** 10.1038/s41598-025-12040-4

**Published:** 2025-07-31

**Authors:** Lara Pfuderer, Hugo Sax, Robert Grass

**Affiliations:** 1 Functional Materials Laboratory, Department of Chemistry and Applied Biosciences, Institute for Chemical- and Bioengineering, ETH Zurich, HCI E 111, Vladimir-Prelog-Weg 1-5/10, 8093 Zurich, Switzerland; 2 Department of Infectious Diseases, Bern University Hospital, University of Bern, Bern, Switzerland

**Keywords:** Pathogen transmission, Surrogate markers, Nanoparticles, Hand hygiene, Disinfectants, Simulation, Infectious diseases, Membrane lipids, Policy and public health in microbiology

## Abstract

Understanding the transmission routes of microbial pathogens is essential for infection prevention and control in healthcare settings. However, using infectious microorganisms to this end is challenging and poses potential risks. We explored alcohol-sensitive DNA-encapsulating lipid nanoparticles (LNP) as surrogate tracers to investigate microbial transmission, including the effect of hand hygiene. LNPs embedded in various synthetic matrices were evaluated under controlled laboratory conditions to identify the optimal formulation. The chosen LNP glycerine and sucrose formulation was subsequently tested in patient care experiments, both with and without hand hygiene conducted according to established standards using alcohol-based hand rub (ABHR). Predefined contact surfaces and body sites were sampled and analysed for LNP integrity through quantitative polymerase chain reaction. The study revealed that the LNP formulation remained stable when dried on a surface. Hand hygiene using ABHR reduced LNP integrity to ≤ 1%. Without hand hygiene, LNPs transferred between surfaces maintained nearly 100% integrity. However, stability decreased during skin-to-skin transfer. In a typical patient care interaction, the study LNP formulation demonstrated a rapid, low-risk, and reliable approach for evaluating short pathogen transmission pathways, including the impact of hand hygiene. It shows promise as a diagnostic tool for assessing the effectiveness of transmission prevention measures in real-life clinical settings.

## Introduction

Healthcare-associated infections (HAIs) are both detrimental and costly. Therefore, preventing HAIs requires a comprehensive approach that addresses all potential transmission routes. Pathogens spread through healthcare providers’ hands^1^, surfaces^2^, air^3^, and water^4^. However, microorganism detection and prevention are challenging. Identifying pathogens on surfaces is difficult due to the need for specialised equipment and handling infectious samples. Surrogate tracers offer a practical alternative for studying transmission pathways.

Effective surrogates should optimally be stable on dry surfaces, transferrable through contact, and susceptible to disinfection processes. Traditional surrogates, such as fluorescent dyes^5^ and the tracing of free^6^ and silica-encapsulated DNA^7–9^, have enhanced our understanding of pathogen transmission, but they are not sensitive to disinfection, limiting their ability to model real-world scenarios involving hand hygiene and surface cleaning.

Lambda phages have been proposed as disinfection-sensitive surrogates^10^; however, their limited stability, biological origin, and challenges related to bacterial contamination reduce their practicability. Their potential reproduction via ubiquitous *Escherichia coli* bacteria also limits their use^11^. An ideal surrogate would model both bacteria and viruses, be non-living, non-toxic, and easy to measure, while having the ability to generate multiple identities (‘barcodes’) for tracking and measuring various sources of contamination simultaneously.

The susceptibility of microbes to ethanolic disinfectants is mainly due to their lipid membranes^12–14^. A phospholipid bilayer, as seen in liposomes, represents the simplest synthetic lipid membrane model. Liposomes and lipid nanoparticles (LNPs), utilised as drug carriers, are non-toxic and can encapsulate nucleic acids for sensitive detection through quantitative polymerase chain reaction (qPCR). Notably, LNPs are used in Moderna’s and Pfizer’s COVID-19 vaccines, which have been approved as safe i.e., by the FDA^15^. Furthermore, synthetic DNA amplicons allow for virtually unlimited barcode generation.

Lipid nanoparticles encapsulating DNA (LNPs) serve as simple, non-replicating models of bacteria or viruses, offering susceptibility to ethanol-based disinfection and easy detection via the encapsulated DNA. While LNPs have been effective in verifying surface disinfection^16^, they are unstable when dried^15,17,18^, unlike bacteria and viruses that remain infectious after partial desiccation^19–22^. This stability is attributed to several stabilising mechanisms, especially the extracellular matrix^23^. Therefore, for LNPs to be suitable surrogates, they must retain stability when dried on surfaces.

This study develops LNP formulations using synthetic matrices to protect them from disintegration. The suitability of these stabilised LNPs as surrogates for microbial pathogens was investigated, focusing on their role in monitoring transmission pathways, including hand hygiene effects.

## Results

### Identification of a suitable LNP formulation

LNP compositions in various synthetic matrices were assessed as microbial pathogen surrogates based on stability when dried, transfer efficiency and integrity. Given the known challenges of drying LNPs^24,25^, stabilisation strategies were explored using sucrose, glycerine, and their combination as simplistic mimics of an extracellular matrix.

**Fig. 1:**
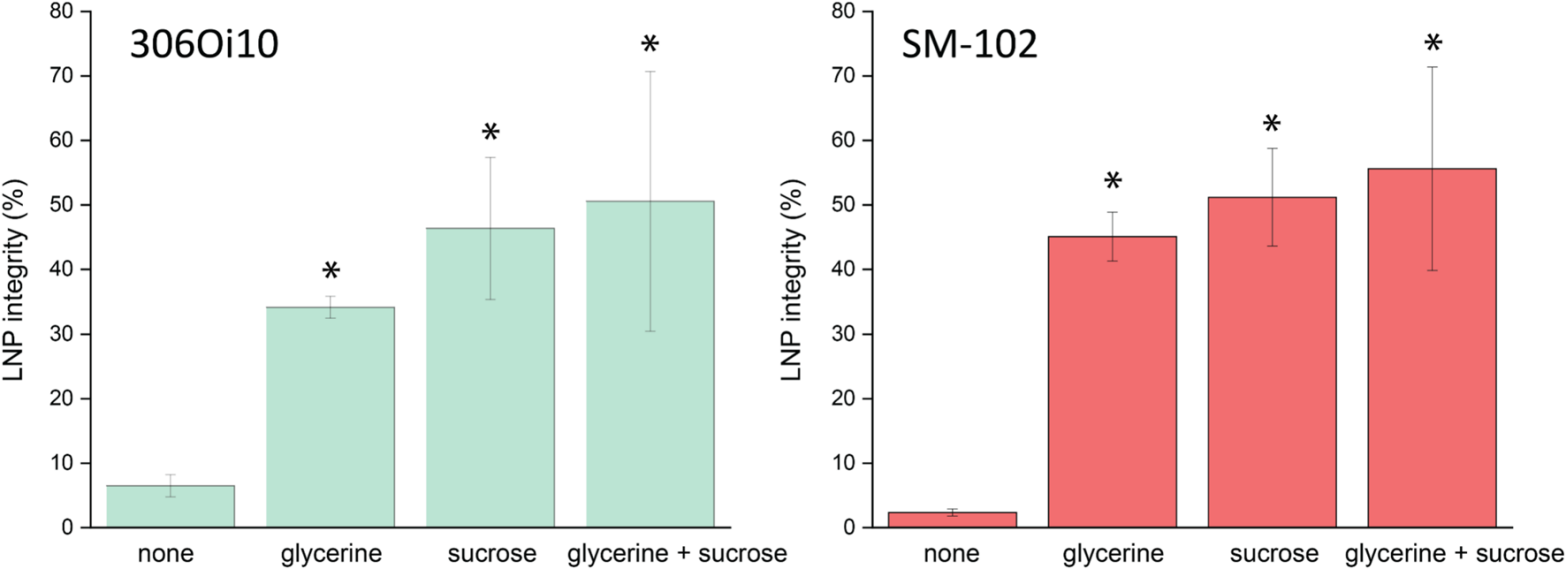
LNP Integrity of two different LNP compositions (306O_i10_ and SM-102) evaluated via integrity PCR when dried on a glass surface with glycerine, sucrose and a combination thereof as synthetic matrices. (*) *p* < 0.05, two sample t-test comparing glycerine, sucrose, or glycerine + sucrose to no synthetic matrix. The error bars show the standard deviation of each measurement, n = 3.

The LNP composition containing SM-102 showed higher *LNP integrity* with a value of 55.6% than the LNP composition containing 306O_i10_ when dried on a surface (Fig. 1). Therefore, only the SM-102 LNP composition was chosen for further experiments. LNPs without a synthetic matrix were unstable when dried on a surface since the *LNP integrity* was below 10% after drying, with values of 2.3% and 6.5% for 306O_i10_ and SM-102, respectively (Fig. 1).

**Fig. 2:**
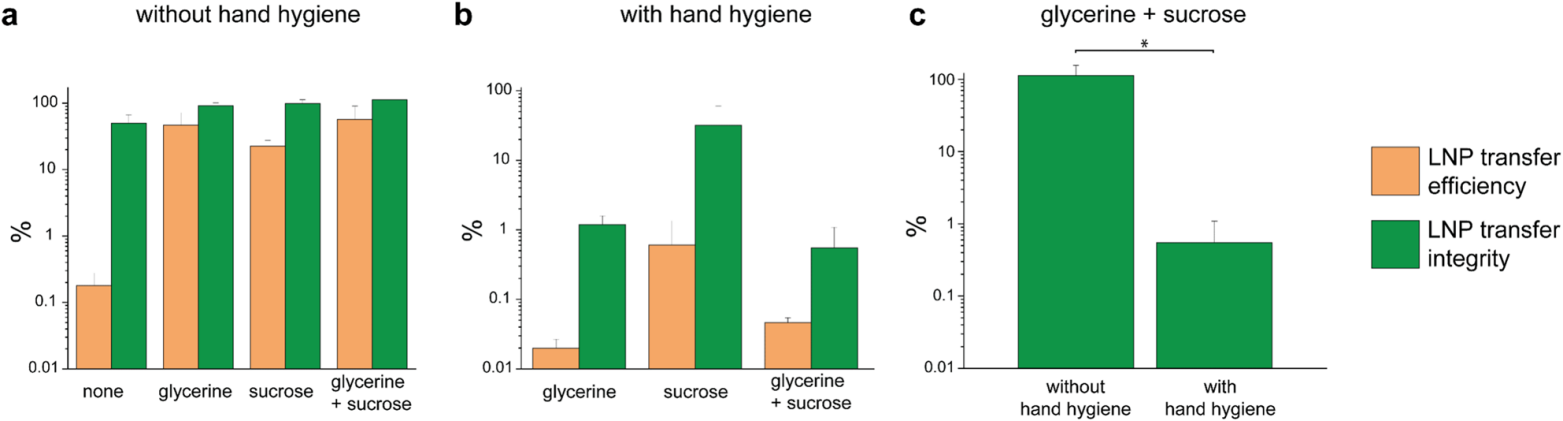
(**a**) *LNP transfer efficiency* (how much DNA gets transferred) and *LNP transfer integrity* (how many particles remain intact upon transfer) on glass slides by gloved index finger without hand hygiene in percent for SM-102 LNPs in glycerine, sucrose and a combination thereof as synthetic matrix. (**b**) *LNP transfer efficiency* and *LNP transfer integrity* on glass slides by gloved index finger with hand hygiene in percent for SM-102 LNPs in glycerine, sucrose and a combination thereof as synthetic matrix. Transfer without matrix was not measured, since *LNP transfer efficiency* was determined to be inefficient already without hand hygiene. (**c**) Comparison of *LNP transfer integrity* in glycerine + sucrose matrix with and without hand hygiene (two sample t-test: (*) *p* < 0.05). The error bars in all figures (**a**, **b**, **c**) show the standard deviation of each measurement, n = 4.

The glycerine-sucrose synthetic matrix showed the highest *LNP transfer efficiency* with 64.8% and *LNP transfer integrity* with 76.8% during transfer without hand hygiene (Fig. 2a). In contrast, transfer without a matrix was highly inefficient, with only ~ 0.2% of DNA being transferred. Consequently, further investigations focused solely on LNPs in a synthetic matrix. *LNP transfer efficiency* with hand hygiene was much lower than without hand hygiene (a and b). This is due to the rubbing action of the hands during hand hygiene, which spreads the DNA over the entire surface of the hands, whereas the transfer to the receiver surface is only via a fingertip. *LNP transfer integrity* decreases after hand hygiene, for example in glycerine where the *LNP transfer integrity* drops from 91.8 to 1.2% with intercurrent hand hygiene (Fig. 2b). The formulation containing sucrose alone partly protected the LNP from disintegration by the disinfectant with a *LNP transfer integrity* of 31.8% (Fig. 2b), which can be explained by the relatively low solubility of sucrose in ethanol^26^. The formulation containing both sucrose and glycerine exhibited a significantly lower *LNP transfer integrity* (≤ 1%, p < 0.05) following hand hygiene compared to no hand hygiene (Fig. 2c).

DNA-loaded LNPs (1 µl) with SM-102, combined with a synthetic matrix of glycerine (10 µl, 100%), sucrose (20 µl, 50 wt%), and external DNA (10 µl, 1 g/l), met the required criteria for LNP transfer efficiency and integrity (Figs. 1 and 2). This formulation was therefore the preferred choice as a disinfection-sensitive pathogen surrogate in *patient care experiments*. We defined a sample with an *LNP transfer integrity* value below 1% as indicative of effective hand hygiene according to these *laboratory experiments*.

### Laboratory-based patient care experiment

**Fig. 3:**
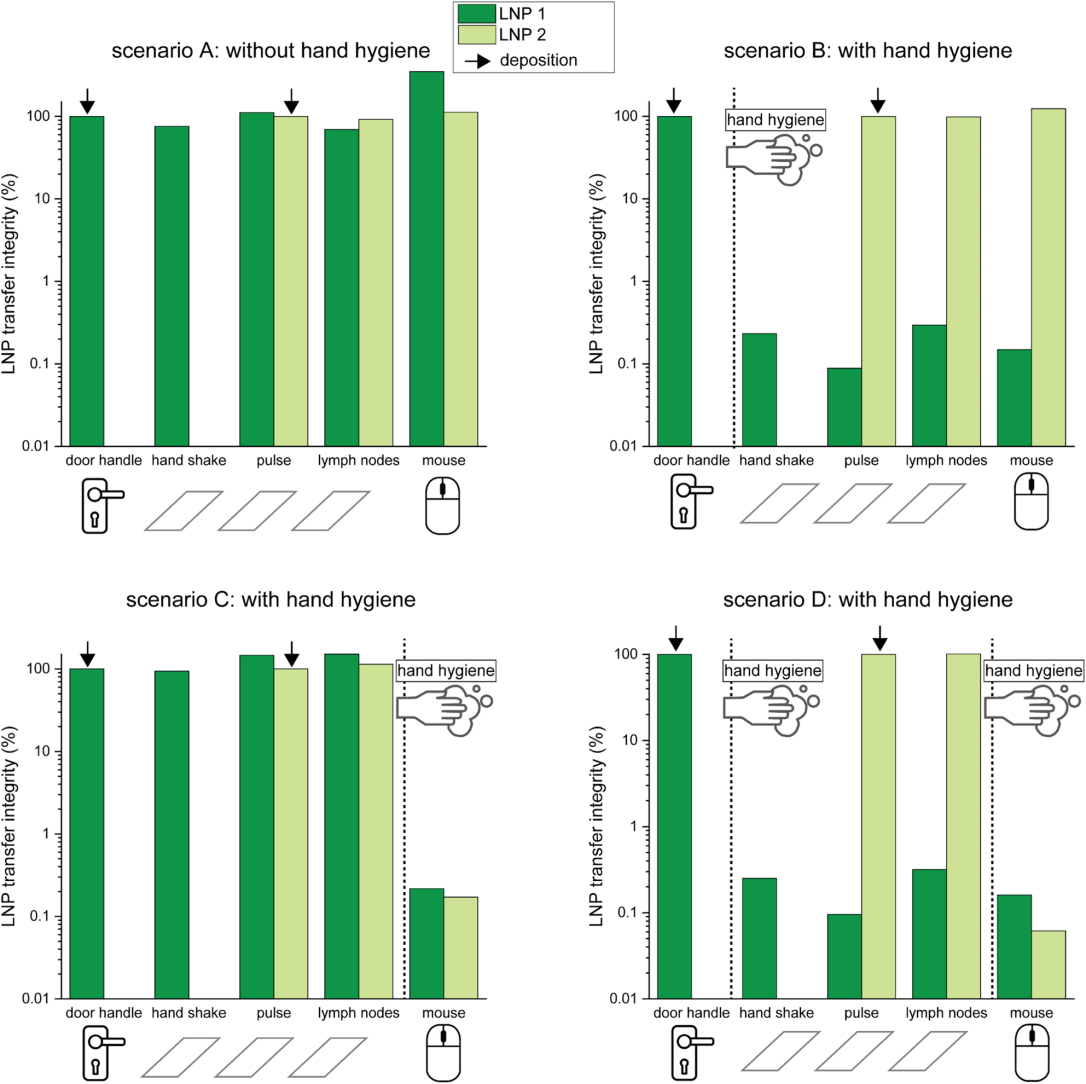
*LNP transfer integrity* measured for 5-step transfer scenarios on coverslips using two LNP barcodes. LNP 1 (dark green) was applied to the door handle, LNP 2 (light green) was applied to the glass slide, representing the patient’s pulse. The healthcare provider first touched the door handle, then the glass slides representing the patient’s hand, pulse, lymph nodes, and finally, the mouse. Each scenario was performed once and the scenarios differ in the occurrence of intercurrent hand hygiene, as indicated by the vertical lines. *LNP transfer integrity* was measured on all surfaces after each scenario.

Figure 3 shows the resulting *LNP transfer integrity* of the *laboratory-based patient care experiment*, where the healthcare provider (HCP) touched coverslips instead of patient body sites. Hand hygiene with ABHR leads to low values of *LNP transfer integrity* of ≤ 1% (0.2% ± 0.1%), while *LNP transfer integrity* remained essentially unchanged without hand hygiene (107% ± 27%). For corresponding *LNP transfer efficiency* results see Figure S1.

### Clinical patient care experiment

**Fig. 4:**
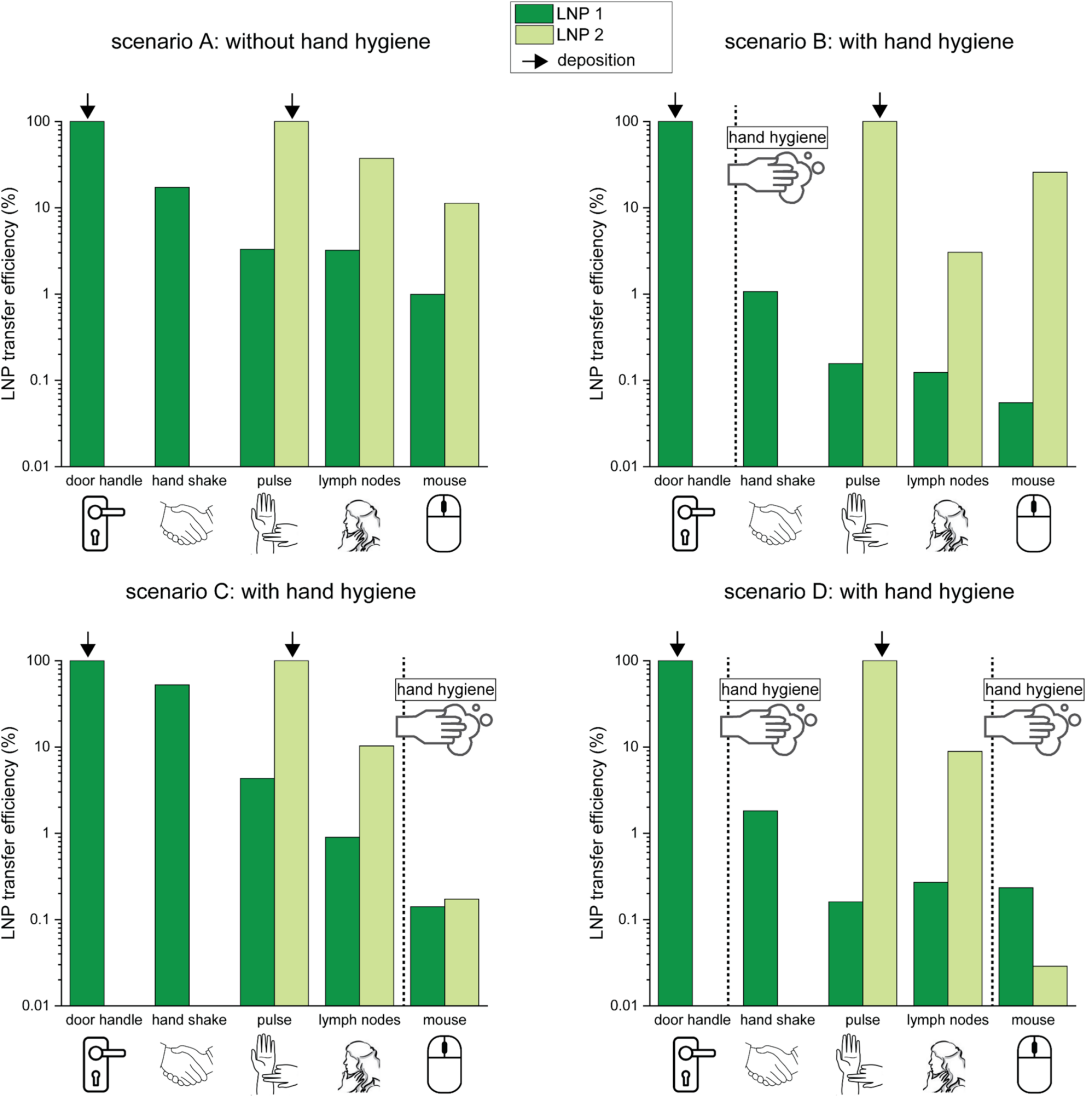
*LNP transfer integrity* measured for 5-step transfer scenarios on patient body sites using two LNP barcodes. LNP 1 (dark green) was applied to the door handle, and LNP 2 (light green) was applied to the patient’s pulse. The healthcare provider first touched the door handle, shook hands with the patient, and then touched the patient’s pulse, lymph nodes, and the mouse. Each scenario was performed once and the scenarios differ by occurrence of intercurrent hand hygiene actions as indicated by vertical lines. *LNP transfer integrity* is measured on all surfaces after each scenario.

Figure 4 shows the resulting *LNP transfer integrity* of the *clinical patient care experiment.* Without intercurrent hand hygiene, *LNP transfer integrity* declined to 68% ± 25% per transfer and with intercurrent hand hygiene to 0.4% ± 0.3%. Notably, in scenarios A and B, the predefined threshold for the effect of hand hygiene of 1% *LNP transfer integrity* was reached after four or three steps of skin-to-skin transfer, with respective values of 0.99% and 0.9%, even in the absence of hand hygiene. For corresponding *LNP transfer efficiency* results see Figure S2.

### Effect of hand washing

After hand washing with water and soap at the end of each scenario, DNA concentration was consistently found to be below the lower limit of detection.

## Discussion

The study aimed to find and test a simple, non-toxic, effective surrogate tracer for microbial pathogens. With its sensitivity to ABHR, LNPs were designed to provide a more realistic model of pathogen transmission compared to existing methods, such as fluorescent dyes and silica-encapsulated DNA. LNPs demonstrate the same low detection limit as silica nanoparticles encapsulating DNA (SPED) due to qPCR^7–9^, which enables tracing through multiple transfer events despite dilution effects, such as those caused by hand rubbing during hand hygiene. The ability to measure two parameters in LNP samples, makes it possible to identify the effect of disinfection, meaning LNP disintegration (*LNP transfer integrity*) independently from the effect of LNP dilution (*LNP transfer efficiency*). To confirm LNP disintegration during hand hygiene, *LNP transfer integrity* is used, while *LNP transfer efficiency* does not allow for the reliable confirmation of hand hygiene, see Figures S1, S2.

Transmission tests without hand hygiene in the *laboratory-based patient care experiment* showed only a small reduction of *LNP integrity*. However, a stepwise reduction of *LNP integrity* occurred in the *clinical patient care experiment* with skin-to-skin interactions. This reduction of *LNP integrity* on skin was unexpected, particularly as it occurred only with skin-to-skin contact transmission. This phenomenon could be attributed to the presence of antimicrobial peptides and lipids on skin^27,28^, which can interact with the lipid membrane of LNPs and compromise their stability^29,30^. Moreover, the LNP disintegration could also be due to different tribology during skin-to-skin transfer as compared to skin-to-surface^31^. A similar reduction in bacterial survival is observed on hands or during skin-to-skin contact^32–34^. The extent to which the LNP model reflects microbiological reality of skin exposure remains to be investigated.

LNPs encapsulating DNA further allow the opportunity to use distinct DNA-sequences. By deploying LNPs encapsulating different barcodes at different sites (see Figs. 3 and 4), it is possible to track multiple sources of contamination in the same experiment. Particularly in a hospital environment, where there are many people working and potentially causing transmission, it is useful to have a method of tracking and measuring different sources of contamination simultaneously.

Due to the decreased stability during skin-to-skin transfer of the proposed LNP formulation only controlled and short transmission scenarios can be reliably investigated. One useful example could be the validation of doffing procedures of personal protective equipment, where the protocol is fixed and there are few skin-to-skin transfers. Another occasion where LNPs could be useful is in testing hand hygiene compliance during a gowning procedure or in investigating fomite-to-glove transfers during surgery.

This study has limitations. We performed only a limited number of transmission experiments and the LNP varied stability, while, however, seeing similar results across these experiments. Further development of the system and comparison with microbial behaviour of various potential pathogens is possible based on the current LNP formulation.

## Conclusion

This study evaluated lipid nanoparticles encapsulating DNA as possible surrogates for microbial pathogens in tracing transmission pathways. LNPs in a synthetic matrix comprising of glycerine and sucrose were shown to have similar properties to microbial pathogens. Namely, they are stable when dried on a surface, they can be transmitted by touch, and they are susceptible to hand hygiene. The designed LNP formulation was successfully used in a short clinical patient care experiment to determine whether efficient hand hygiene has taken place. However, the LNP integrity was reduced during skin-to-skin transfer. In conclusion, the proposed LNP formulation has been shown to be useful for investigating and monitoring short transmission routes, including hand hygiene, of microbial pathogens in real-life healthcare settings. Further development and testing to mimic specific pathogen behaviour is warranted.

## Methods

The study had three phases: (1) *laboratory experiments* to identify the optimal LNP composition (types of LNPs) and formulation (LNP-matrix combination), (2) testing the selected formulation in a *laboratory-based patient care experiment* with touching inanimate surfaces with bare hands, and (3) a *clinical patient care experiment* including touching skin of participants representing patients^35^.

### Synthesis of LNP compositions

Based on dedicated literature^15,16^ and our experience, we selected two LNP compositions for the *laboratory experiments*, one composition containing the lipidoid 306O_i10_, which has been previously investigated^16,36^, and another composition containing SM-102 as an ionisable lipid, which is known for its increased stability and is close to the formulation used for the Moderna COVID-19 vaccine Spikevax^15^.

First, an organic phase containing the lipids and an aqueous phase containing the DNA were prepared. This procedure was the same for both selected LNP compositions. For particles containing the lipid 306O_i10_^16^, DOPE (1,2-dioleoyl-snglycero-3-phosphoethanolamine, Avanti), cholesterol (Sigma-Aldrich), C14-PEG1000 (1,2-dimyristoyl-snglycero-3-phosphoethanolamine-N-[methoxy(polyethylene glycol)-1000] (ammonium salt), Avanti), and 306O_i10_ (tetrakis(8-methylnonyl) 3,3′,3″,3‴-(((methylazanediyl) *bis*(propane-3,1-diyl))*bis*(azanetriyl))tetrapropionate) were dissolved in 90% ethanol and 10% 10 nM citrate buffer at molar ratios of 16:46.5:2.5:35. For LNPs with the lipid SM-102, DSPC (1,2-Distearoyl-sn-glycero-3-phosphocholine, Adipogen), cholesterol (Sigma-Aldrich), C14-PEG1000 (1,2-dimyristoyl-snglycero-3-phosphoethanolamine-N-[methoxy(polyethylene glycol)-1000] (ammonium salt), Avanti) and SM-102 (9-Heptadecanyl (8-(2-hydroxyethyl)[6-oxo-6-(undecyloxy)hexyl]amino) octanoate, Cayman) were dissolved in ethanol at molar ratios 10:38.5:1.5:50.

The aqueous phase was prepared to comprise of an annealed DNA amplicon (Microsynth AG, for sequences see Supplementary Table S1), 10 mM Tris base buffer (pH = 7), and 10 nM citrate buffer, with a final DNA concentration of 1 g/l. Both phases were preheated to 37 °C, and the ethanol phase was added dropwise to the aqueous phase in a 3:1 volumetric ratio and mixed by rapid pipette mixing. After mixing, twice the volume of 300 Mm NaCl buffer (pH 4) was added and the LNPs were incubated at 37 °C for 1 h, shaking at 450 rpm. The particles (2 g/l) were stored at 4 °C.

### Sample analysis

Samples were collected from surfaces by rolling a pre-moistened (milliQ water) swab (PCR-Nasal swab, MFS-96000BQ, Meidke Gene) over an area of approximately 3 cm^2^. The swab was placed in a clean Eppendorf tube, cut to fit the cap, and 200 µl milliQ water was added. Analysis was conducted as described elsewhere^16^.

**Fig. 5:**
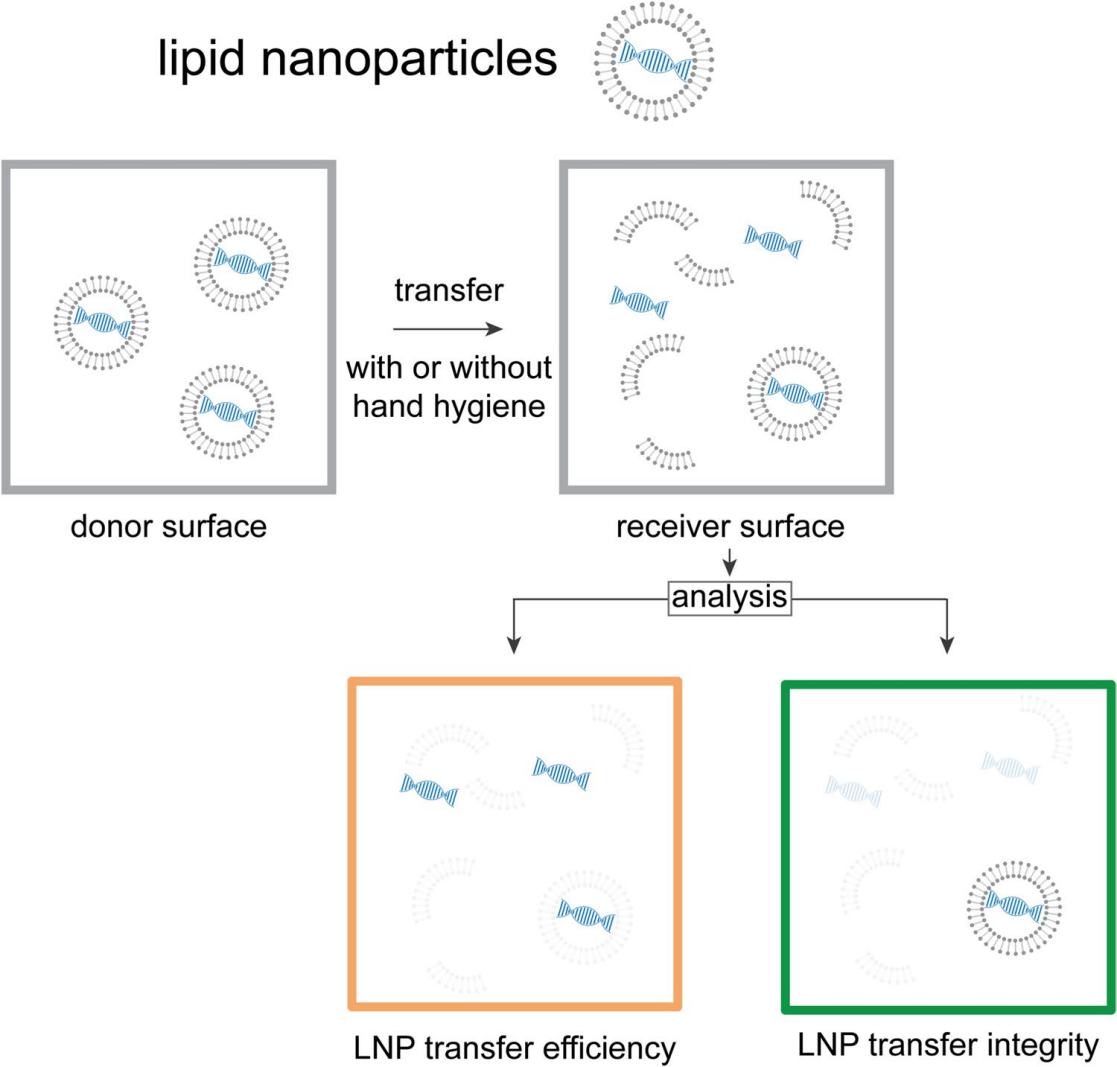
Schematic representation of different measurable parameters using LNPs, namely *LNP transfer efficiency* and *LNP transfer integrity*. *LNP transfer efficiency* quantifies the proportion of DNA successfully moved from the donor to the receiver surface by the transfer process and *LNP transfer integrity* is a measure of degradation of LNPs when transferred from the donor to the receiver surface with or without intermittent hand hygiene. *LNP transfer integrity* is expected to decrease with intermittent hand hygiene, because LNPs are known to disintegrate when in contact with ABHR.

Two parameters were determined from each sample: (1) total DNA and (2) LNP integrity (Fig. 5). Thereby, LNP integrity represents viability qPCR in viruses^37,38^.

*LNP transfer efficiency* quantifies the proportion of DNA successfully moved from the donor to the receiver surface by the transfer process. It is calculated from the difference in cycle threshold between the total DNA measured on the receiver surface and the total DNA remaining on the donor surface measured after transfer, expressed as:

1 $$\Delta Ct\left( {LNP\;transfer\;efficiency} \right) = Ct(total\;DNA_{{receiver}} ) - Ct(total\;DNA_{{donor}} )$$

2 $$LNP\;transfer\;efficiency\left( \% \right) = 100 \cdot \frac{1}{{2^{{\Delta Ct\left( {LNP\;transfer\;efficiency} \right)}} }}$$

Here, the DNA quantity measured on the receiver surface is $total \;DNA_{receiver}$ and $total \;DNA_{donor}$ is the DNA quantity measured on the donor surface. The values are given in cycle thresholds $Ct$, representing the number of PCR amplification cycles necessary to obtain a signal.

To assess the *LNP integrity* on a surface, we measured the proportion of DNA encapsulated within intact LNPs. The sample was divided into two portions: one was treated with Benzonase, an enzyme that degrades unencapsulated DNA while leaving DNA within intact LNPs unaffected, and the other portion remained untreated, containing both free and encapsulated DNA. *LNP integrity* was then calculated from the difference in cycle thresholds between encapsulated DNA (from the treated sample) and the total DNA (from the untreated sample), expressed as:

3 $$\Delta Ct\left( {LNP\;integrity} \right) =  Ct\left( {encapsulated\;DNA_{{treated\;sample}} } \right) - Ct\left( {total\;DNA_{{untreated\;sample}} } \right)$$

4 $$LNP\;integrity\left( \% \right) = 100 \cdot \frac{1}{{2^{{\Delta Ct\left( {LNP\;integrity} \right)}} }}$$

The resulting value represents the fraction of DNA that remained protected within LNPs expressed as percentages. To evaluate $LNP\; transfer\; integrity$, i.e., a measure of degradation of LNPs when transferred from the donor to the receiver surce with or without intermittent hand hygiene, we determined the change in $LNP\; integrity\;$ between the donor and receiver surfaces, indicating how well the LNPs preserved their structural integrity during the transfer. $LNP\; transfer \;integrity$ is expected to decrease with intermittent hand hygiene, because LNPs are known to disintegrate when in contact with alcohol-based hand rub (ABHR)^16^.

5 $$\Delta \Delta Ct\left( {LNP\;transfer\;integrity} \right) = \Delta Ct(LNP\;integrity_{{receiver}} ) - \Delta Ct(LNP\;integrity_{{donor}} )$$

6 $$LNP\;transfer\;integrity\left( \% \right) = 100 \cdot \frac{1}{{2^{{\Delta \Delta Ct\left( {LNP\;transfer\;integrity} \right)}} }}$$

In all transfer experiments, the measurements on every surface, including the donor surfaces, were taken after transfer, as swabbing the donor surface before contact would have removed a significant amount of LNPs and, consequently, compromised the results.

### Identification of a suitable LNP formulation

To ensure enduring resistance to desiccation on dry surfaces, *LNP integrity* was assessed. Each LNP composition (1 µl, 2 g/l) was applied to a surface and dried either alone or within synthetic matrices: glycerine (10 µl, 100%), sucrose solution (20 µl, 50 wt% in water), or a sucrose-glycerine combination (20 µl + 10 µl), at 1000 times the LNP mass, yielding eight formulations. External DNA (10 µl, 1 g/l) with a distinct sequence (see Supplementary Table S1)was added to control for LNP reformation. Additional components (chitosan, dextran, agarose, methyl cellulose) had no further effect.

To serve as surrogates for microbial pathogens, LNPs must transfer efficiently between surfaces while remaining intact. *LNP transfer efficiency* and *integrity* were assessed by applying LNPs in synthetic matrices (glycerine, sucrose, or both) to an initial coverslip (borosilicate glass, Roth), then transferring them via a gloved fingertip (nitrile, semper care) to a second clean coverslip. High values for both parameters indicate effective transfer without disintegration.

The susceptibility to disinfection is defined as LNP disintegration upon exposure to ABHR. To assess this, gloved hands were disinfected with Sterillium med (85 g ethanol/100 g, Hartmann Group, Heidenheim) using a standardised three-step method^29^ between touching the first and second coverslip.

### Laboratory-based patient care experiment

To assess LNPs as pathogen surrogates, a typical patient examination was modelled using two distinct LNP barcodes. An experimenter (LP) acted as the HCP, while clean coverslips (borosilicate glass, Roth) represented patient body sites. The door handle was contaminated with LNP-1, and the coverslip representing the patient’s left wrist with LNP-2.

The experiment began with the HCP opening the door, contaminating the bare right hand with LNP-1., then touching the coverslip representing the patient’s right hand with right thenar, followed by touching the coverslip representing the left wrist (LNP-2) with right index and middle fingers. Next, the coverslip representing the patient’s submandibular lymph nodes with both index and middle fingers was touched before interacting with a computer mouse to simulate data entry.

This experiment was repeated four times, with and without hand hygiene performed in distinct instances following the WHO *My Five Moments for Hand Hygiene* (Fig. 4) resulting in four different scenarios^39^. Hand hygiene was executed using a palmful of Sterillium med (85 g ethanol/100 g, Hartmann Group, Heidenheim) for 15 s according to a validated three-step method^40^. After each scenario, samples were collected from the door handle, ‘patient hand’, ‘wrist’ and ‘lymph node’ coverslips, and computer mouse before cleaning.

### Clinical patient care experiment

To assess the impact of human skin, the transmission experiment was repeated using actual patient body sites instead of coverslips but following the same procedure. Again, door handle and patient pulse were contaminated with LNP-1 and LNP-2, respectively. An experienced medical doctor (HS) acted as the HCP, while lab members served as patients. The experiment was conducted four times over two days, with different patient actors, with and without hand hygiene, resulting in four different scenarios. After each scenario, samples were collected from the door handle, patient’s right hand, left wrist, lymph nodes, and computer mouse before cleaning.

### Effect of hand washing

After each scenario of the clinical patient care experiment, the HCP washed hands following the WHO ‘How to handwash’ poster^27^ using liquid soap (sodium laureth sulfate, fresh soap, MBudget). Both hands were then sampled for LNP contamination.

## Ethical approval

The Ethics Board of the Canton of Zurich reviewed the study protocol and formally waved the necessity for a full ethics review based on the Swiss Law on Research on Humans [BASEC-Nr. Req-2024-01187]. All procedures were performed according to local (Swiss) guidelines and regulations and study participants gave oral consent, after being given information about the lipid nanoparticle surrogates.

## Supplementary Information

Supplementary Information.

## Data Availability

All data generated or analysed during this study are included in this published article and its supplementary information files.
