# Supplementary material for: DNA lipid nanoparticles as alcohol-sensitive surrogates to trace microbial transmission and monitor hand hygiene
Source: Sci Rep. 2025 Jul 31;15:27965. doi: 10.1038/s41598-025-12040-4 (PMC12314018; doi:10.1038/s41598-025-12040-4)
Supplement: Supplementary file 1 — Supplementary Information. [file 41598_2025_12040_MOESM1_ESM.docx]

**SUPPLEMENTARY MATERIAL for**

DNA Lipid Nanoparticles as alcohol-sensitive surrogates to trace microbial transmission and monitor hand hygiene

Lara Pfuderer (1), Hugo Sax (2), Robert Grass (1)

1. Functional Materials Laboratory, Department of Chemistry and Applied Biosciences, ETH Zurich, Zurich, Switzerland
2. Department of Infectious Diseases, Bern University Hospital, University of Bern, Bern, Switzerland

**Supplementary Figure A1. Lipid nanoparticle (LNP) transfer efficiency in the laboratory-based patient care experiment**
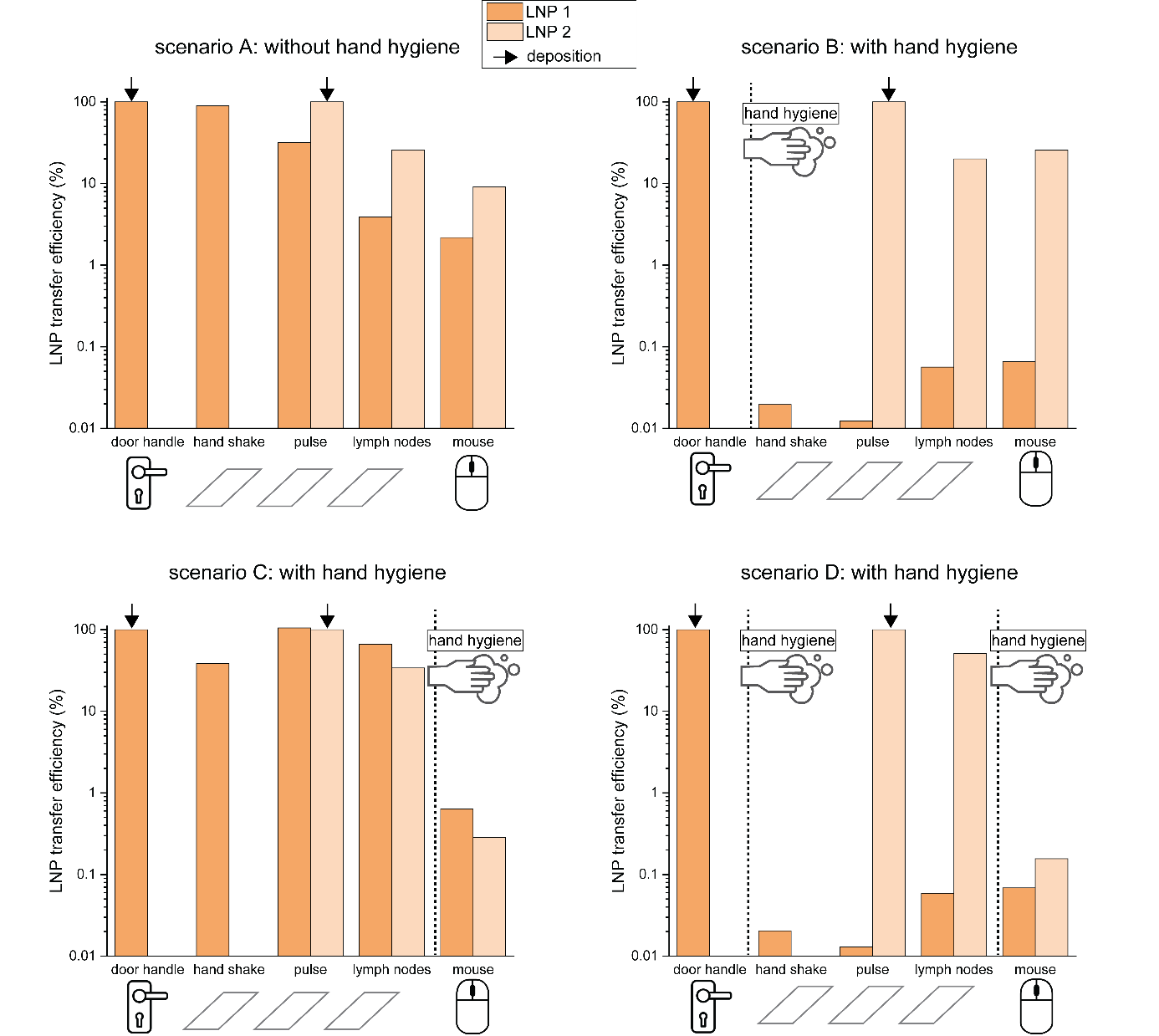


**Figure A1 Legend:** *LNP transfer efficiency* measured for 5-step transfer scenarios on coverslips using two LNP barcodes. LNP 1 (dark orange) was applied to the door handle, LNP 2 (light orange) was applied to the glass slide, representing the patient’s pulse. The healthcare provider first touched the door handle, then touched the glass slides representing the patient’s hand, pulse, lymph nodes, and finally, the mouse. Each scenario was performed once and the scenarios differ in the occurrence of intercurrent hand hygiene, as indicated by the vertical lines. *LNP transfer efficiency* was measured on all surfaces after each scenario.

**Supplementary Figure A2.** **Lipid nanoparticle (LNP) transfer efficiency in the clinical patient care experiment**


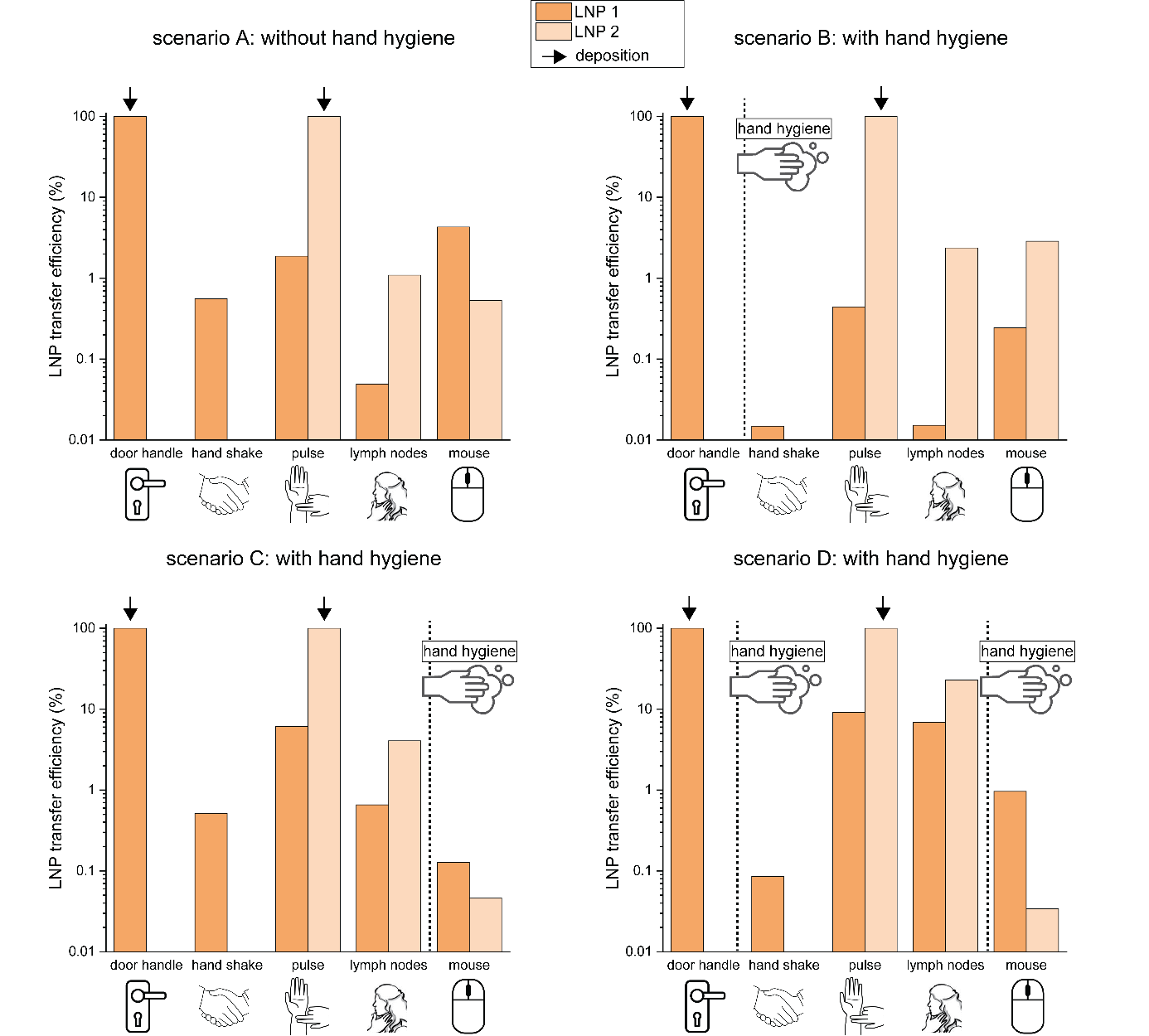


**Figure A2 Legend:** *LNP transfer efficiency* measured for 5-step transfer scenarios on patient body sites using two LNP barcodes. LNP1 (dark orange) was applied to the door handle, and LNP2 (light orange) was applied to the patient’s pulse. The healthcare provider first touched the door handle, shook hands with the patient, and then touched the patient’s pulse, lymph nodes, and the mouse. Each scenario was performed once and the scenarios differ by occurrence of intercurrent hand hygiene actions as indicated by vertical lines. *LNP transfer efficiency* is measured on all surfaces after each scenario.

**Supplementary Table A1. List of sequences and primers.**

| **Sequence name** | **Sequence 5’-3’** |
| --- | --- |
| GM-06-S4 (barcode 1) | TTCTCTGCCCTTACGTTTATCTTAAGGGCCGGTC  CACCAGTTGAACACGAACAAACCTCTTT |
| GM-06-S4  primer forward | CTCTGCCCTTACGTTTATC |
| GM-06-S4  primer reverse | AGAGGTTTGTTCGTGTTC |
| kumquat3  (barcode 2) | GACGATTCAAAGACGTTGCTGATATGCTTCAAGATTATATT  CCCAGCCTTAAAATGGCTAACGACG ACTCCGGTTCGGTTTCCTGTGATAGCTCCTCTCAACAACTCTC |
| kumquat3  primer forward | GACGATTCAAAGACGTTGCTG |
| kumquat3  primer reverse | GAGAGTTGTTGAGAGGAGCT |
| External DNA | AACTGCACTGATGACCGCACTGACTCTATGCCGACTAAT  GTACAGGCTTGTGATCGCGTCGAGTA |
